# Inter-organisational Cooperation Oriented Towards Sustainability Involving SMEs: a Systematic Literature Review

**DOI:** 10.1007/s13132-023-01196-x

**Published:** 2023-03-14

**Authors:** Nathalia Suchek, Mário Franco

**Affiliations:** 1 Department of Management and Economics & NECE Research Center in Business Sciences, University of Beira Interior, Estrada do Sineiro, 6200-209 Covilhã, Portugal; 2 Management and Economics Department, CEFAGE-UBI Research Center, University of Beira Interior, Estrada do Sineiro, 6200-209 Covilhã, Portugal

**Keywords:** Sustainability, Circular economy, Cooperation, Networks, SLR, SME

## Abstract

Small and medium-sized enterprises (SME) are under increasing pressure to implement sustainability practices and collaborate in sustainable development. To do so, they can choose to collaborate with other organisations in order to overcome the challenges and barriers found in moving towards sustainability. Sustainability issues in SMEs have been discussed in the last two decades, but the knowledge on the inter-organisational collaboration towards sustainability remains dispersed. In this sense, this paper aims to answer the question: what is known about inter-organisational cooperation oriented towards sustainability involving SMEs? A systematic literature review (SLR) was carried out using 55 articles available on *Web of Science* (WoS) aiming to understand the processes of inter-organisational relations towards sustainability involving SMEs, simultaneously addressing the emergence of the circular economy. As a result, the articles were organised into four groups, namely (1) cooperation for sustainability promoted by government initiatives, (2) effects of inter-organisational cooperation for sustainability, (3) process of cooperation oriented towards sustainability, and (4) start of discussions on cooperation for the circular economy. A framework is presented with an overview of the evolution of the field, highlighting the main factors and outcomes related to inter-organisational cooperation involving SMEs for sustainability. The review provides theoretical implications as future research avenues for academics and scholars as well as practical implications for entrepreneurs, managers and policy-makers.

## Introduction

The current lifestyle and its association with negative environmental and social impacts have occupied more and more space in discussions. The concept of sustainable development first introduced by the Brundtland Report indicates that “sustainable development is development that meets the needs of the present without compromising the ability of future generations to meet their own needs” (Brundtland, 1987). Over time, the concept has attracted increasing attention, and concern about sustainable development has intensified, especially after the publication of the United Nations’ 2030 Agenda, which introduced 17 goals based on the three dimensions of sustainable development, i.e. the economic, environmental and social dimensions (United Nations, 2015).

In this sense, the concept of circular economy (CE) emerges as a strategy to operationalise sustainable development (Geissdoerfer et al., 2017), as an alternative to the linear economic system based on “take, make, dispose”, characterised by the priority given to economic objectives with little consideration for ecological and social concerns (Sauvé et al., 2016). CE is defined as “an economic system that replaces the ‘end-of-life’ concept with reducing, alternatively reusing, recycling and recovering materials in production/distribution and consumption processes” (Kirchherr et al., 2017, p. 229). This way, CE has implications at micro (products, companies and consumers), meso (eco-industrial parks) and macro (cities, regions, nations and beyond) levels, being enabled novel business models and responsible consumers (Kirchherr et al., 2017).

In this regard, private sector initiatives are expected to contribute to sustainable development through investments, solutions development and business practices adoption (Frey & Sabbatino, 2018; Rashed & Shah, 2021; Scheyvens et al., 2016). In this way, small and medium-sized enterprises (SME) are key actors since SMEs account for 99.8% of firms in the European Union and 66.6% of jobs (European Commission, 2020). Besides that, it is highlighted that SMEs cause 64% of the environmental impact (Constantinos et al., 2010). Therefore, SMEs’ introduction of sustainable practices is key to achieving economic, social and environmental goals.

Although some SMEs consider investment in sustainable practices an additional burden (Hoogendoorn et al., 2015), those adopting a sustainable orientation can reach competitive advantages arising from their efforts (Jansson et al., 2017). Business people involved in sustainability are motivated not only by legal requirements and environmental concerns but by cost efficiency, eco-efficiency, creating value by taking advantage of opportunities generated by sustainable demands, greater staff retention and improved company image (Bos-Brouwers, 2010; Revell et al., 2010). Therefore, SMEs can be the target for (sustainable) investment by large firms, create SME networks in sustainable markets or become sustainable suppliers in global supply chains (Moore & Manring, 2009).

Innovations of an incremental or radical nature are observed from different strategic behaviour. Through such behaviour, SMEs introduce innovations in processes (for example, cleaner production, eco-efficiency and logistics), organisation (for example, environmental management systems, supply chain management and reflexive innovation process) and products (for example, ecological design, life-cycle analysis and fair trade products) (Klewitz & Hansen, 2014).

Whether in implementing the CE or in moving to sustainability in general, SMEs face challenges. The barriers to engaging in introducing sustainable practices include a lack of interest from suppliers and customers, lack of capital and increased costs, lack of government support, lack of technical know-how, administrative workload, lack of information and the firm’s environmental culture, among others (Revell et al., 2010; Rizos et al., 2016). In the case of the CE, another challenge is the fact of it being a recent topic with few practical examples able to demonstrate its implementation.

To overcome the barriers in transiting to sustainability and the recognised limited resources of SMEs, firms can choose to act in cooperation with other organisations. Cooperation with other actors such as customers, suppliers, knowledge institutions and even peers allows firms to share resources, access knowledge and form relationships leading to the development of sustainable innovations (Bos-Brouwers, 2010). Through financial and organisational efficiency, SME networks allow the development of technology and markets essential to achieve sustainable development (Moore & Manring, 2009). Networks let firms identify best practices and provide a learning environment, creating conditions for the development of new products and services (Jenkins, 2009). Increased interaction between the different network members and establishing collaborative approaches can help firms overcome barriers and successfully integrate circularity in their strategies (Eikelenboom & de Jong, 2021).

According to Prashar and Sunder (2020), researchers’ interest in firms’ sustainable development issues is recent, particularly since 2016. In that period, the effects of business networks on small firms’ sustainable performance began to be analysed. To the authors’ knowledge, to date, there are no systematic literature reviews (SLR) on the topic, and the knowledge around inter-organisational cooperation oriented towards sustainability involving SMEs remains dispersed. Indeed, previous SLR report that cooperation and partnership within supply networks are strategic alternatives to mitigate the impact of certain eco-innovation determinants in manufacturing SMEs that deserve deeper studies (de Pacheco et al., 2017), while the SRL on inter-organisational collaboration and SMEs’ innovation does not approach sustainability issues (Zahoor & Al-Tabbaa, 2020). Considering the need to understand the processes of inter-organisational relations involving SMEs to overcome perceived barriers and achieve sustainable results and paying special attention to the emergence of the CE as a way to operationalise and achieve those objectives, an SLR was carried out to answer the following research question: *What is known about inter-organisational cooperation oriented towards sustainability involving SMEs?* The use of the RSL is justified by the fact that this is a methodology that allows an in-depth understanding of previous research on the topic, identifying gaps and avenues for future research (Kraus et al., 2022),

This SLR covered 55 publications indexed on Web of Science dealing with inter-organisational cooperation oriented towards sustainability involving SMEs. It was organised in four groups: (1) cooperation for sustainability promoted by government initiatives, (2) effects of inter-organisational cooperation for sustainability, (3) process of cooperation oriented towards sustainability, and (4) start of discussions on cooperation for the circular economy. From a theoretical perspective, our study incorporates the field of knowledge dedicated to analysing the determinants for sustainability and innovation in SMEs, especially the case of inter-organisational cooperation, by simultaneously covering these three subjects (i.e. SMEs, sustainability and inter-organisational cooperation). The literature is organised into four groups, and an overview of the evolution of the topic was presented, highlighting the important factors in the process of inter-organisational cooperation and the main potential outcomes. Our article also provides insights for entrepreneurs, managers and policy-makers as well as future research avenues for academics and scholars.

Next, the “Methodology” section describes the methodology in detail. The “Results**”** section presents the systematic literature review results, namely the descriptive data of the sample of articles and a description of the studies identified by groups. Finally, the “Discussion**”** section presents the main conclusions and suggestions for future research.

## Methodology

An SLR can be used to establish the grounding for new research or to summarise what is currently known or unknown about a topic (Carver et al., 2013). In the first phase, a gap was identified in the research on inter-organisational cooperation oriented towards sustainability involving SMEs, which gave rise to the research question presented above.

Subsequently, the terms to be included in the search, the scientific database to be used and the inclusion and exclusion criteria were defined in a second phase. To identify publications dealing simultaneously with sustainability, the circular economy and inter-organisational collaborations involving SMEs, it was opted for search through keywords by topics (including titles, abstracts and keywords), using the following terms: “circular econom*” OR “sustainab*” AND “collab*” OR “cooperat*” OR “partner*” OR “alliance*” OR “network*” AND “SME*” OR “small and medium sized enterprise*” OR “small firm*” OR “small business” OR “small enterprise*”. The Web of Science (WoS) was used as a source of scientific literature. The WoS covers thousands of journals. It is generally considered the most comprehensive database for scientific work, becoming a reference in the scientific community (Dahlander & Gann, 2010: Paul & Criado, 2020). Although some journals are not in the database, the WOS typically includes the most prominent journals in a research field (Dahlander & Gann, 2010). Table 1 shows the inclusion and exclusion criteria defined, adapted from the SLR carried out by Johnson and Schaltegger (2016) about sustainability management tools in SMEs.

**Table 1:** Inclusion and exclusion criteria

Criterion	Reason for inclusion/exclusion
Inclusion criteria	
Articles written in English	Most academic journals are published in English
Peer-reviewed articles	To ensure the quality and consistency of the analysis, only peer-reviewed articles were included
Articles in the categories of “business”, “management” and “environmental sciences” in Web of Science	To ensure the focus of the paper on SME management and sustainability
Exclusion criteria	
Theoretical and conceptual articles	To ensure the empirical basis of the research
Articles not addressing matters of sustainability	To ensure that the term “sustainability” was applied to environmental and social issues rather than only economic ones
Articles not addressing matters of inter-organisational cooperation	To ensure the research focus on relations between organisations
Articles not including SMEs in their analysis	To ensure the research focus on SMEs

In the third phase, it was conducted the search and data collection (April 2021). We do not apply temporal filters to obtain a sample that reflects the entire evolution of the area. After entering the search terms in the WoS, we applied three more inclusion criteria. We selected only articles in English as this is the academic community’s lingua franca, and most journals are published in this language (Johnson & Schaltegger, 2016; Kraus et al., 2022). We also selected only peer-reviewed articles to ensure the quality and consistency of the analysis, as these are subjected to rigorous peer review processes (Zahoor & Al-Tabbaa, 2020). To ensure the focus of research on SME management, we selected articles published in journals associated with the categories “business” and “management”. Additionally, we selected articles published in journals associated with the “environmental studies” category, as recent reviews show that articles focusing on sustainability in business have been published in environmental-oriented journals (He et al., 2018). After applying the inclusion criteria, the sample contained 322 articles.

Next, exclusion criteria were applied from scanning the titles and abstracts. The full text was analysed when the abstract did not provide enough information to assess whether the article should be excluded. The focus of this SLR is on empirical evidence; therefore, theoretical and conceptual articles were excluded from the sample. To ensure the focus of this study is on inter-organisational cooperation oriented towards sustainability involving SMEs, we applied three additional exclusion criteria. Articles using the term “sustainability” with a meaning other than sustainable development (e.g. articles using the term sustainability in the strictly economic sense disregarding environmental and social dimensions), articles that did not contribute significantly to the analysis of inter-organisational relationships and articles that provide findings only for large enterprises were excluded. The SLR analysed a final sample of 55 articles.

The fourth phase consisted in summarising the evidence and interpreting the findings. First, a descriptive analysis of the sample was carried out, and then a content analysis of the papers under analysis was performed. The literature review was organised by conceptual order (four groups), also following a chronological order to illustrate the development of the literature.

## Results

In this session, we present the results of the descriptive analysis of the sample and the content analysis conducted, which gave rise to four groups of studies.

### Descriptive Data

**Fig. 1:**
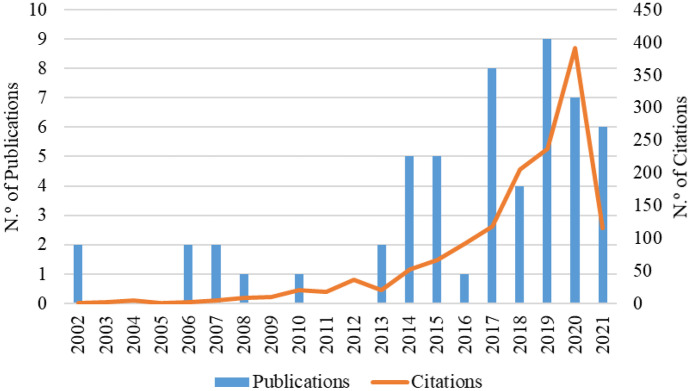
Evolution of publications and citations per year

The descriptive data of the sample allowed a more detailed analysis of the evolution of the topic. Figure 1 shows that the first publications date from 2002, being very occasional until 2013. The number of publications increased from 2014, but growth was not linear. The evolution of publications and citations per year shows that interest in the subject of inter-organisational cooperation for sustainability began to gain prominence, particularly from 2013, and continued to grow. Eighty-five percent of the articles in the sample were published between 2013 and 2020. The evolution of publications may be a reflection of international initiatives in favour of sustainability and which in turn have received great recognition, such as the United Nations Conference on Sustainable Development held in Brazil, also known as Rio 20+ (United Nations, 2012), and the Agenda 2030 that introduced the 17 sustainable development goals (United Nations, 2015). This peaked at 9 publications in 2019, as in 2020, the number fell to 7, which may result from the effects of the COVID-19 pandemic in academic research.

Regarding the journals publishing on the topic studied (Table 2), the ones with most articles are *Journal of Cleaner Production* and *Sustainability*, with 20 and 9, respectively. *Business Strategy and the Environment* and *Resources, Conservation and Recycling* published two articles each. It is noted that two journals with most publications are included in the category of “*environmental sciences*”, considered inter-disciplinary, which may indicate that study of inter-organisational cooperation for sustainability involving SMEs is not yet well-defined in business sciences.

**Table 2:** Number of publications and total citations per journal

Journal	Number of publications	Total citations
Journal of Cleaner Production	20	539
Sustainability	9	74
Business Strategy and the Environment	3	424
Resources, Conservation and Recycling	3	45
International Journal of Entrepreneurial Behaviour & Research	2	17
Environmental Engineering and Management Journal	2	39
European Journal of Innovation Management	2	1
Technological Forecasting and Social Change	2	36
Service Industries Journal	1	11
R&D Management	1	18
Foresight and STI Governance	1	1
World Journal of Entrepreneurship Management and Sustainable Development	1	11
Corporate Social Responsibility and Environmental Management	1	0
Global Business Review	1	0
Social Responsibility Journal	1	7
Journal of Manufacturing Technology Management	1	30
Organization & Environment	1	0
International Journal of Technology Management	1	80
Business Ethics: A European Review	1	11
Journal of Business Ethics	1	45

**Fig. 2:**
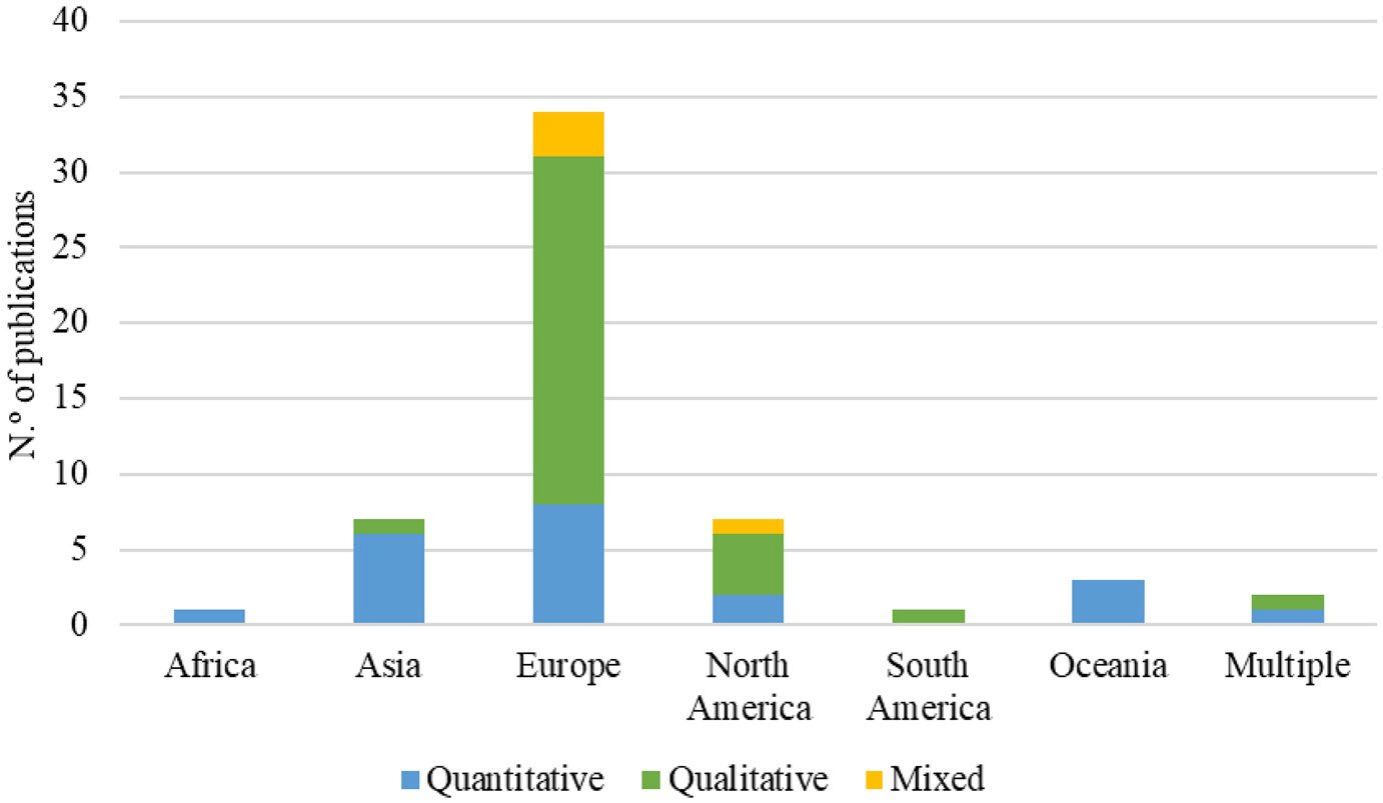
Regions studied and methodologies applied

Figure 2 shows the regions that were the focus of study in publications, as well as the methodology used. The majority of studies concern Europe, followed by North America and Asia. On the other hand, areas such as Africa, South America and Oceania are under-explored, presenting few studies in countries in these regions. These results indicate greater attention to inter-organisational cooperation oriented towards sustainability involving SMEs in developed countries and the Global North, probably due to their advanced position in terms of discourse on sustainability compared to developing countries and the Global South. As for methodology, most studies adopt a qualitative approach.

### Content Analysis

Four groups of studies are identified through content analysis: (1) cooperation for sustainability promoted by government initiatives, (2) effects of inter-organisational cooperation for sustainability, (3) process of cooperation oriented towards sustainability and (4) start of discussions on cooperation for the circular economy. Following the studies are described in each group.

#### Cooperation for Sustainability Promoted by Government Initiatives

The first articles dealing with cooperation oriented towards sustainability involving SMEs are cases of government initiatives and local authority projects following political agendas. These projects present different objectives, such as implementing environmental management systems, minimising waste, preventing pollution, recycling and others, where firms’ economic objectives and results are valued above environmental and social aspects.

Right at the beginning, from the projects that aimed the diffusion of Eco-Management and Audit Scheme (EMAS) through promoting contacts and information and experience exchanges, Biondi et al. (2002) found that cooperation and networking among SMEs and stakeholders are the most effective way to support environmental innovation. Three networks were found, i.e. recurrent transitions and long-term relations, which, when overlapping, influence the adoption of environmental innovations, namely, business networks, regulatory networks and firms’ knowledge networks. Networks are important when beginning a process of adopting innovations (searching for information), at the implementation stage (the more radical the innovation, the more necessary is specialist support), and in consolidation and continuous efficient use of innovation. Cooperation with other firms operating in the same geographical area and/or the same industrial district can help in achieving competitive advantages, as well as cooperation with competitors (coopetition) and with the supply chain.

In the Canadian programme focused on preventing pollution, Granek and Hassanali (2006) found that reliable business networks are essential to gain access to the market and that the involvement of non-profit organisations in the project is welcomed by SMEs since they ensure confidentiality. Regarding local authorities’ role, Von Malmborg (2007) underlined that these could act as teachers or tutors to stimulate learning practices in networks to develop regional sustainable development. Especially regarding teachers’ role, they probably have a greater potential to contribute to the innovation system in the long term, favouring network collaboration between various actors who remain once projects end.

Studies also highlighted the positive outcomes of projects based on partnerships between different actors and SMEs. Phillips et al. (2002) presented the case of a waste minimisation project that produced impressive results in terms of financial economies and training for SMEs. Ackroyd et al. (2006) presented a project in the UK focused on spreading information about environmental conformity and best practices, which resulted in significant cost economies, waste minimisation and energy and water efficiency for the SMEs involved.

Bos-Brouwers (2010) studied the factors influencing the translation of sustainable innovation into practice in SMEs participating in a project for innovation and corporate sustainable development. Cooperation with stakeholders, namely customers, suppliers, knowledge institutions, local government, commercial associations, knowledge networks, design companies, peers, consultants and also a case of a joint venture with international firms are cited in the sustainable innovation process. More recently, Spiesberger and Schönbeck (2019) studied government incentives based on financing vouchers for cooperation between SMEs and research institutions, concluding that this is an effective instrument to stimulate environmental innovation.

#### Effects of Cooperation Oriented Towards Sustainability

In this group are gathered the studies that analyse the effects and outcomes of collaboration. These studies mainly indicate the influence that inter-organisational cooperation have on establishing sustainable practices and innovations in SMEs.

Collins et al. (2007) compared the adoption of sustainable practices between members and non-members of a sustainable business network, observing that members of sustainable business networks demonstrated greater awareness of environmental issues than companies that are not part of such networks. Furthermore, the adoption of sustainable practices is more positively related to SMEs than to large companies, suggesting that networks have a stronger impact on organisational learning in SMEs. Virtual cooperation was reported by Camisón (2008), who carried out a case study of a Spanish network based on a digital warehouse. Results indicate the network’s contribution to new knowledge creation and dissemination and to the configuration of a strong regional supply of advanced knowledge-based services for environmental adaptation in SMEs. This network’s environmental learning also influences environmental and economic performances.

In a programme to promote cleaner production in supply chains in Mexico, Van Hoof (2014) also recognise network relations as facilitating organisational learning. Networking within suppliers allowed the formation of new commercial relations and social contacts in which representatives of other suppliers are considered an important source of information and exchange of experience. Van Hoof and Thiell (2014) investigate how collaborative capability contributes to achieving cleaner production goals within supply chains and networks, founding that this capability results in connection with sustainable supply chain initiatives, design and implementation of environmental projects. Silva and Figueiredo (2020) also argue that cooperating is one of the five practices stimulating sustainability, both internally and throughout the supply chain, with the potential to stimulate the learning process to promote sustainability.

Benito-Hernández et al. (2016) found a positive relationship between cooperative relations with firms’ external stakeholders and activities of environmental protection. This indicates that firms maintain and improve their cooperative relations through networks with universities, competitors, suppliers and legislators, and customers also spend more on environmental protection than others.

Studies indicate positive effects of cooperation on introducing sustainable innovations (Aka, 2019; Frey et al., 2013; Halme & Korpela, 2013; Jun et al., 2021). From the resource point of view, Halme and Korpela (2013) indicated that cooperation in R&D with clients or other stakeholders is seen as a fundamental resource to make up for the lack of knowledge in the sector and acquire capital and reputation, allowing the development and commercialisation of innovations. The cases analysed engaged in active R&D cooperation with customers or other stakeholders to compensate for SME scarce resources. In the green economy sector, Frey et al. (2013) also found that cooperation is one determinant of innovation. Standing out was collaboration with research centres and universities to obtain experience and specific knowledge, being considered reliable partners.

In terms of green marketing innovations, Kumar (2015) showed that small firms related to the strategic partnership demonstrate that working in close collaboration with business partners creates better management of premises, generates economy, increases productivity and improves environmental performance. In turn, Aka (2019) indicated that the development of sustainable innovations, from the approach of co-construction and parallel association of actors, is an interactive, social and synchronous process, where the interactions between actors occur almost simultaneously.

Although most studies indicate the positive effect of cooperation on sustainable innovations, in Pakistan, Jun et al. (2021) could not confirm that external partnership and cooperation contribute to green innovations. The authors argue that this is because the lack of partners with whom to cooperate towards this type of innovation in this context.

The studies do not focus only on the environmental dimension of sustainability. Kraus et al. (2017) concluded that both SMEs with high network involvement and low involvement can find paths to successful social performance. However, even those with low involvement use resource leverage such as arranging with other firms to give each other indications in order to save on marketing costs and use connections with other firms to increase provision economically. Also, a study by Hassan et al. (2019) showed that managerial networks impact safety training, employee involvement, safety rules and procedures and safety promotion policies in SMEs. Dawar and Singh (2020) confirmed that partnerships contribute effectively to corporate social responsibility in India.

Studies also highlight cooperation as a key factor in entering into more sustainable sectors and establishing new sustainable business models. Jernström et al. (2017) found that collaboration with the supply chain is an important factor for SMEs becoming part of the Finnish bio-economy sector. According to Long et al. (2018), collaboration with other actors in the supply chain is necessary to find appropriate suppliers willing to provide supplies that are sustainable to some extent, to reach investors willing to examine alternative business approaches and to promote involvement with clients and consumers to inform and educate them, generally through co-creation.

The more destined to increase private benefit through innovative sustainable business models based on direct relations with the final customer and on the strategic partnership, the more likely these practices are to increase their legitimacy. And the more the innovative, sustainable business model increases its legitimation within normative and cultural-cognitive institutions, the more it will lead to imitation dynamics that initiate the transformation of those institutions (Gasbarro et al., 2018).

#### The Process of Cooperation Oriented Towards Sustainability

Various factors influencing the cooperation process were observed by researchers in the area. An important aspect highlighted is leadership. Boiral et al. (2014) point out that leaders, who they classify as “post-conventional”, are important for SMEs’ environmental commitment, as they exert pressure on suppliers and other stakeholders regarding environmental issues. Vătămănescu et al. (2017) observe that entrepreneurial orientation towards sustainability positively influences network marketing practices, whether in the context of emerging or planned strategies. Lewis et al. (2015) investigate the attitudes of SME owners-managers in collaborating with other firms. The gender and age of owner-managers influence the decision to collaborate, with women and younger adults being more receptive to this practice. On the other hand, firms that collaborate do not show differences in their answers to environmental questions than those that do not collaborate.

Another factor highlighted is the formality of the collaborations. Lewis et al. (2015) have found that most SMEs engaging in collaboration with other organisations did so informally. Testa et al. (2017) observed that the lack of internal resources to react to external pressure related to environmental aspects was overcome by the SMEs following an informal agreement with competitors and a formal agreement with the local chamber of commerce. Such contracts allow clients’ needs to be met with reduced costs and effort. Looser and Wehrmeyer (2015) observed that for Swiss SMEs, collaboration with other stakeholders for social responsibility objectives depends critically on informal contracts. In this way, transition costs are reduced, and more sustainable supply chains are established due to shorter transport distances.

Especially in informal agreements, trust is a fundamental factor. Lee (2019) emphasises the need for a trusting relationship between partner organisations that is simultaneously voluntary and viable. The author did not find a relation between autonomous collaborative activities and green activities among firms of a similar size, meaning that mutual autonomous activities between groups of different sizes are more important. Cutaia et al. (2015) also observe that the lack of cooperation and trust between industries were barriers found in implementing a virtual platform aiming to promote industrial symbiosis.

Thomas et al. (2021) found that stakeholders with non-contractual bonds with SMEs undertake green innovations, including universities and research centres, public administrations, competitors and the community. On the other hand, no effects of contractual bonds with clients, suppliers and investors were found. In addition, public administrations exert a negative influence, i.e. they seem to hinder SMEs about green innovations.

Regarding the objectives of the actors involved in the collaboration, and from the perspective of new sustainable business models, Karlsson et al. (2017) found that effective cooperation is essential in partnership projects where the partners have different responsibilities, motives and interests. Special prominence is given to public-private networks, the need for a long-term perspective that recognises the importance of environmental and social benefits as contributing to possible financial profit and prudent selection of partners. Wadin et al. (2017) studied an alliance between a multinational and an SME for innovation in the business model. The multinational drew maximum benefit from the coded knowledge about new technology but neglected important elements for the business model. On the other hand, the SME needed investment and could expand its market through collaboration. In turn, Russo and Schena (2021) observe that those sustainability-oriented partnerships where the objectives of ambidexterity are balanced reveal better performance. Alliances that seek sustainability aims that are complex and difficult to implement by firms individually should involve sufficiently different partners in order to have complementary resources.

Studying green technology start-ups, Pakura (2020) proposed that open innovation induces risks depending on the source of knowledge and that different sourcing strategies and knowledge sources depend on the firm’s development and the stage of technological innovation. At the first stage, support platforms are more prominent. In contrast, in the second and third stages, those of developing and commercialising technology, established firms play a fundamental role as partners of open innovation.

Since organisational learning is one of the effects of the inter-organisational cooperation, Johnson (2017) studied the capacity of sustainability-oriented SMEs to acquire and develop the explicit knowledge necessary for an environmental management systems and related tools. Three forms of cooperation were identified (participation in a network, strategic alliances and sustainability communities) and differences between initiating and advanced firms (level of expertise) to acquire knowledge related to sustainability. Some authors have dedicated to the study of the absorptive capacity of SMEs, such as Aboelmaged and Hashem (2019) that found that collaboration has a mediating impact on the relation between absorptive capacity and the adoption of green innovation and Hau (2019) that found that absorptive capacity moderates the mediating role of reduced production time in the relation between the diversity of SMEs’ external R&D technology cooperation network and the respective reduction in greenhouse gas emissions and energy savings.

Regarding the actors involved in inter-organisational cooperation, Kundurpi et al. (2021) analysed the role and challenges of intermediaries such as government, traditional and sustainability-oriented business networks, NGOs and other firms. According to the authors, government functions consist of incentives for resource efficiency and financial support for small businesses. Traditional business networks have the function of facilitating learning, networks and business growth, while sustainability-oriented networks offer practical and personalised guidance for action, benchmarks and monitoring, peer support and accreditation/certification. NGOs can give support in the initial phase of setting up and growing and spreading sustainability-oriented practices. Finally, other firms can help share best practices and collaborate in forming sustainable supply chains. As for cooperative relations between SMEs and NGOs, Harangozó and Zilahy (2015) find that SMEs do not usually form relations with non-profit organisations as they do not consider that interaction beneficial. Stekelorum et al. (2020), in turn, observe that cooperating with NGOs can help to develop social requirements in SMEs’ supply chains, strengthening the influence of corporate social responsibility in defining these requirements.

Still, according to Kanda et al. (2018), intermediaries’ activities consist of forecasting and mapping, gathering and spreading information, stimulating networks and partnerships, prototyping and piloting, technical consultancy, resource mobilisation, commercialisation, branding and legitimation.

Concerning cooperation between SMEs and higher education institutions, Halila and Tell (2013) present the results of a project involving a university and SMEs to obtain ISO 14001 certification. The study highlights that SMEs can turn to learning networks to overcome barriers to implementing environmental management systems. Sáez-Martínez et al. (2014) analyse SMEs in 27 European Union countries and find that cooperation with universities and research institutions directly affects the development of eco-innovations. In addition, cooperation positively moderates the effect of policy regulations and factors on the supply side on entrepreneurs’ likelihood of developing this type of innovation. Jones and Corral de Zubielqui (2016) examine university-firm interactions and the results of innovation and firm performance in sustainability-oriented innovations in Australia, finding that firms tend to opt for generic links, i.e. using human resource transfer, scientific publications and ideas from informal sources in universities to access knowledge. Furthermore, according to Emilsson et al. (2020), collaboration with academia can support product assessment, educate participants and mediate between different stakeholders who generally have different visions of sustainable innovation.

#### Start of Discussions On Cooperation for the CE

Studies on cooperation involving SMEs and other organisations with goals related to the CE are recent. The main motivations and barriers are observed, clearly showing cooperation as part of the new circular business models.

According to Ünal et al. (2019), one of the three components of the circular business model is the value network, i.e. effective collaboration based on trust between suppliers and manufacturers, ensuring long-term relationships and their participation in the CE. This allows the development of new competencies through innovation and adoption of new technology (product innovation and process innovation), using the skills and resources of the value chain network to face the challenges. Moreover, Ünal et al. (2019) argue that value creation in circular business models involves the support of all partners to develop awareness and new skills, making the business model more viable for all supply chain actors and establishing effective communication with suppliers, retailers and managers of material at the end of its useful life (such as the waste industry), as well as with all the other actors involved.

Brown et al. (2019) also studied why firms collaborate in order to reach circular oriented innovations. The motivations for collaborating vary between intrinsic (activities pursued due to their own interest) and extrinsic (activities that bring external rewards or avoid punishment), originating at the business or personal level. The tactical-operational motives quoted include focusing on resources, a context for experimenting and collaboration to operationalise the business model.

Patricio et al. (2018) attempted to understand SMEs’ challenges and motivations related to industrial symbiosis. Economic gains, as well as environmental performance and marketing reasons, were presented as the main motivations for firms to engage in IS partnerships. At the same time, the lack of time and knowledge were indicated as the main barriers. Rincón-Moreno et al. (2020) also identified challenges in establishing industrial symbiosis favouring the CE: organisational management, waste management and resource management. The first refers to problems in forming synergistic relations for the social dynamics of different actors, involving problems related to technological assistance and fiscality of information. The second recognises the lack of leadership of waste management firms in SMEs, which leads to a model where waste is seen as a resource. The third concerns little synchronisation in developing guidelines or procedures to manage resources, as well as highly centralised logistics, which makes it difficult to support the process of initiating resource partnerships in a potential local circular system. In this process, leaders have a very important role in stimulating the development of network interactions to integrate circularity in the business strategy (Eikelenboom & de Jong, 2021).

Finally, the process of collaboration is also studied. Brown et al. (2020) analyse how firms collaborate towards circular-oriented innovations. The authors differentiate collaboration processes between incremental and radical innovations, with the former having a more traditional approach to collaboration while the latter presents a portfolio approach.

## Discussion

This paper aimed to answer the research question regarding what is known about inter-organisational cooperation oriented towards sustainability involving SMEs. Existing scientific articles were gathered with 55 being included in the sample for analysis. As is the case of research on inter-organisational collaboration and innovation in SMEs (Zahoor & Al-Tabbaa, 2020), research on sustainability-oriented inter-organisational cooperation involving SMEs have received less attention in leading journals in the field of entrepreneurship and small business. Moreover, Europe gained prominence as the main context studied. On the other hand, while quantitative studies over-represent research on inter-organisational collaboration and innovation in SMEs, when the focus is on sustainability, we see the opposite, with a predominance of qualitative studies. The main journal publishing on the topic is the *Journal of Cleaner Production*, similar to corporate eco-innovation research (He et al., 2018). Moreover, while the study of inter-organisational collaboration for innovation and the determinants of eco-innovation in SMEs gained prominence from 2010 onwards (de Pacheco et al., 2017; Zahoor & Al-Tabbaa, 2020), sustainability-oriented inter-organisational cooperation involving SMEs started to evolve consistently a few years later, since 2013.

**Fig. 3:**
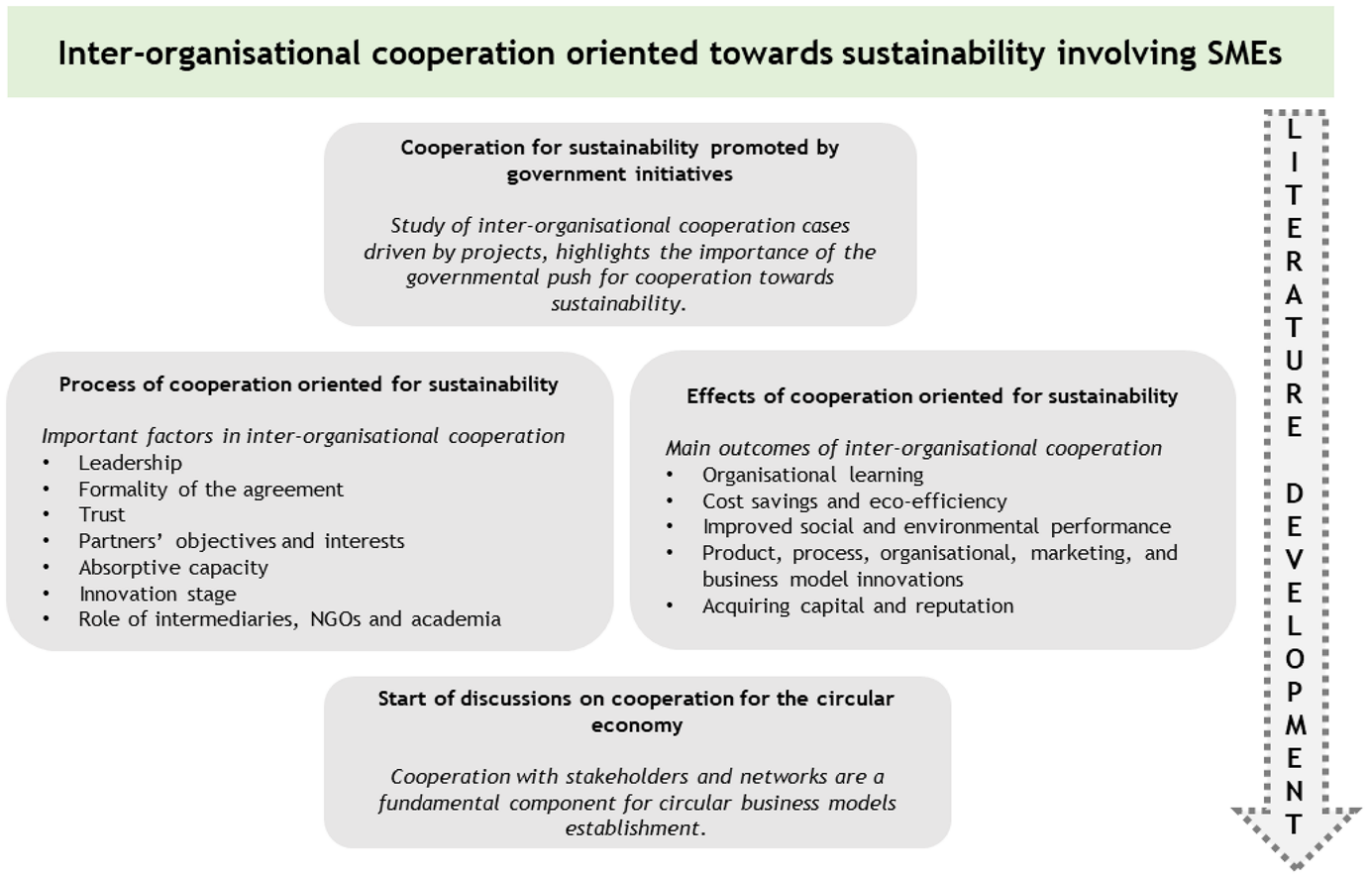
Inter-organisational cooperation oriented towards sustainability involving SMEs

The SLR was organised in four groups. The first, cooperation for sustainability promoted by government initiatives, shows that in a first period, SMEs that engaged in inter-organisational cooperation to achieve sustainability objectives did so through government initiative projects with mostly economic aims, through eco-efficiency and resource productivity. The second group joined studies dealing with the effects of inter-organisational cooperation for sustainability as a fundamental factor in developing innovations in products, processes and the business model. This group also highlights the results achieved through the collaboration, including organisational learning, financial economies, greater awareness of environmental issues and greater expenditure on environmental protection, among others. The third group includes studies analysing the factors influencing the process of sustainability-oriented cooperation. Finally, the fourth group deals with the start of discussions on cooperation towards the CE involving SMEs, pointing out the main motivations and barriers related to cooperation, as well as its incorporation in the circular business model. Figure 3 represents the literature related to inter-organisational cooperation towards sustainability involving SMEs, positioning these four groups according to the development of the literature.

## Conclusion

This paper presents a SLR of the empirical evidence around the field of research interested in studying inter-organisational cooperation from the perspective of SMEs with a focus on sustainability outcomes. This is a fragmented topic of study so far; therefore, our study provides several contributions from the identification, systematisation and overview of relevant literature.

From a theoretical perspective, previous studies providing a review of the intersection between SMEs research, inter-organisational cooperation, and sustainability are lacking. Literature reviews involving SMEs and sustainability often report partnerships and collaborations as relevant determinants, but these are not the central aim of these investigations (de Pacheco et al., 2017; Suchek et al., 2022). Furthermore, Zahoor and Al-Tabbaa’s (2020) review that focuses on the relationship between inter-organisational collaboration and innovation in SMEs does not address sustainability issues involved in this relationship. Therefore, our study incorporates the field of knowledge dedicated to analysing the determinants for sustainability and innovation in SMEs, especially the case of inter-organisational cooperation, by simultaneously covering these three subjects (i.e. SMEs, sustainability and inter-organisational cooperation). We have organised the literature into four groups, and, as illustrated in Fig. 3, we present an overview of the evolution of the topic, highlighting the important factors in the process of inter-organisational cooperation and the main outcomes. We also present the state of empirical research in methodological terms, which presents contributions to the methodological evolution of the field.

From a practical perspective, by identifying the main factors involved in the process of inter-organisational cooperation, as well as highlighting the potential outcomes of cooperation, our article provides contributions for entrepreneurs, managers and policy-makers. It could be used as a basis for decision-making for managers and stakeholders aiming to cooperate to achieve sustainable outcomes and meet the current global requirements. It could also be useful for policy-makers to develop successful mechanisms to promote cooperation for sustainability involving SMEs and different stakeholders.

Future research should position cooperation as a central focus when analysing sustainability in SMEs and further understand the relationships from each actor’s perspective, individually and in greater depth. It is also necessary to understand what other factors are important in the cooperation process and in what circumstances the factors identified are more or less relevant. It is fundamental to specify the conditions of cooperation in order to understand the results that contribute to sustainable development. In this way, it will be possible to orient SMEs regarding questions they should consider in the cooperation process and what results can be achieved in the quest for sustainability. The descriptive results of the articles highlight that despite the number of studies increasing in recent years, most were carried out in the European context. It is important to study other regions so that results can be compared. Indeed, cooperation to achieve sustainability objectives involving SMEs is fundamental in developing countries, where SMEs have even more limited resources. The results of the SLR revealed a predominance of qualitative studies. Therefore, future research can combine the important factors and potential outcomes identified in this paper and develop quantitative studies with more comprehensive and representative samples. Furthermore, the circular economy has gained increasing attention from scholars and policy-makers as a way to operationalise sustainability, so future studies within the circular economy framework are also needed.

Finally, using only one database of scientific production and excluding articles not written in English and not indexed in the categories of Web of Science referred to are considered limitations of the study, thereby excluding scientific publications which may be relevant.
